# Cell Penetrating Peptoids (CPPos): Synthesis of a Small Combinatorial Library by Using IRORI MiniKans

**DOI:** 10.3390/ph5121265

**Published:** 2012-11-23

**Authors:** Dominik K. Kölmel, Daniel Fürniss, Steven Susanto, Andrea Lauer, Clemens Grabher, Stefan Bräse, Ute Schepers

**Affiliations:** 1 Institute of Organic Chemistry, Karlsruhe Institute of Technology (KIT), Fritz Haber Weg 6, 76131 Karlsruhe, Germany; Email: dominik.koelmel@kit.edu (D.K.K.); daniel.fuerniss@kit.edu (D.F.); braese@kit.edu (S.B.); 2 Institute of Toxicology and Genetics, Karlsruhe Institute of Technology (KIT), Hermann-von-Helmholtz-Platz 1, 76344 Eggenstein-Leopoldshafen, Germany; Email: steven.susanto@student.kit.edu(S.S.); andrea.lauer@student.kit.edu (A.L.); clemens.grabher@kit.edu (C.G.)

**Keywords:** combinatorial chemistry, split and mix, library, peptoid, IRORI, sub-monomer, cell penetrating

## Abstract

Cell penetrating peptoids (CPPos) are potent mimics of the corresponding cell penetrating peptides (CPPs). The synthesis of diverse oligomeric libraries that display a variety of backbone scaffolds and side-chain appendages are a very promising source of novel CPPos, which can be used to either target different cellular organelles or even different tissues and organs. In this study we established the submonomer-based solid phase synthesis of a “proof of principle” peptoid library in IRORI MiniKans to expand the amount for phenotypic high throughput screens of CPPos. The library consisting of tetrameric peptoids [oligo(*N*-alkylglycines)] was established on Rink amide resin in a split and mix approach with hydrophilic and hydrophobic peptoid side chains. All CPPos of the presented library were labeled with rhodamine B to allow for the monitoring of cellular uptake by fluorescent confocal microscopy. Eventually, all the purified peptoids were subjected to live cell imaging to screen for CPPos with organelle specificity. While highly charged CPPos enter the cells by endocytosis with subsequent endosomal release, critical levels of lipophilicity allow other CPPos to specifically localize to mitochondria once a certain lipophilicity threshold is reached.

## 1. Introduction

For more than a decade, polycationic cell penetrating peptides (CPPs) have been well established as molecular transporters for intracellular drug delivery or as probes for bioconjugation [1,2,3,4]. They have been either used as covalently bound moieties for a variety of cargos such as peptides and proteins, DNA and RNA, nanoparticles and small molecules or as complexing agents for nucleic acids due to their electrostatic properties [5,6]. However, CPPs often display an inefficient bioavailability due to their proteolysis and opsonization in the presence of serum. Many approaches such as the retroinversion of the CPP sequence or the conversion to β-peptides have been developed to increase the stability by making them resistant to proteases and even more efficient transporters [7,8,9,10]. Surprisingly, treatment of cells with these peptides, however, resulted in a severely increased toxicity or in long term accumulation in the kidney [7,11]. Besides several others, polycationic cell penetrating peptoids (CPPos; *N*-substituted oligoglycines) have been synthesized by several means as protease resistant and less toxic mimetics of CPPs [12,13,14,15,16,17,18,19,20,21,22,23,24,25]. Although the CPPos do not always resemble the conformational constraints of the respective peptides, as the chirality of the α-carbon is lost, they can be modified by a large variety of functional side chains offering a toolkit for the development of artificial cell penetrating moieties. By introducing side chains, which include conformational constraints, the structure of the peptoids can be influenced to retain the properties of the corresponding CPPs. Eventually, they will provide sources for specific organelle- and organ-targeting transporters. For the discovery of novel targeting CPPos, microscopy based phenotypic high-throughput screening seems to be a potent technique. However, this often requires large and chemically diverse compound libraries. In recent years, peptoid synthesis has been established on solid phase to allow for the combinatorial synthesis of highly diverse libraries of peptoids [14,16,17,25,26,27,28,29]. The synthesis of diverse peptoid scaffolds is usually performed by the so called submonomer approach through microwave-assisted amide bond formation and subsequent nucleophilic substitution with primary amines or *via* heterocyclic halomethyl carboxylic building blocks [25].

**Figure 1:**
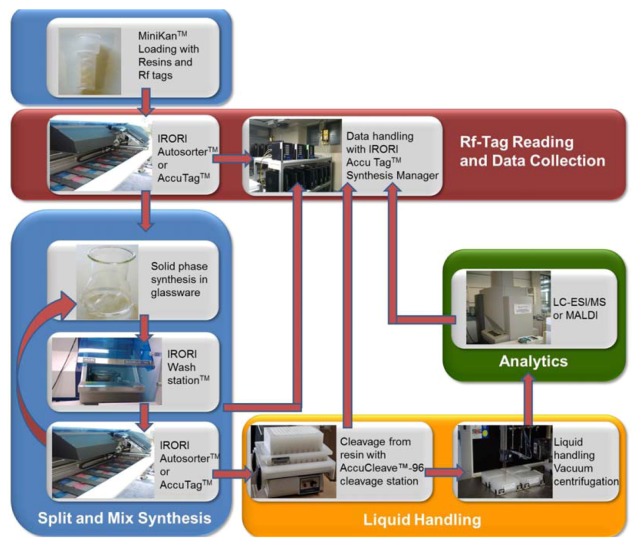
Schematic representation of the IRORI based solid phase chemistry of split and mix compound libraries. Depending on the size of the library the sorting can be performed manually by using the Accu TagTM Reader for up to 100 Kans or in an automated fashion by the Autosorter 10Kx (up to 10,000 Kans).

Recently, the latter approach was used for the synthesis of a one bead-one compound library of peptoids comprising a theoretical diversity of about 16,000 peptoids with a representation of 87% of the theoretical compounds [25]. Although this kind of library is suitable for the screening with different biological targets to mine for structurally diverse ligands, it is not applicable for the screening of targeting CPPos as they have to be released from the respective bead to enter cells. For the application in phenotypic cellular screens, larger amounts of the peptoids are necessary, which cannot be achieved by the one bead-one compound technology. Automated peptoid synthesis can overcome these problems with yields. However, a conventional peptoid synthesizer based on a peptide synthesizer does not allow for the synthesis of large split and mix libraries. The IRORI technology is a commercially available format of nano- up to macro-reactors that allows for maximizing the benefits of split and mix synthesis while enabling the automated production of larger amounts of single compounds per reactor [30,31,32,33,34,35,36] (Figure 1). This technology supports the synthesis of large and chemically diverse libraries by the use of directed sorting technology based on glass coated radiofrequency (Rf) tags embedded in the reactors. Chemistry in the reactors or so called Kans can be performed by combining the Kans in standard glassware. Initially, Kans are loaded with the appropriate resin and the Rf tags prior to capping. Depending on the size of the library, the split and mix process can be managed by the IRORI AccuTag Synthesis Manager and sorting either by an Rf-tag reader or the fully automated sorting procedure with the IRORI AutoSort 10Kx instrument, which is designed to accept MiniBlocks harbouring up to 96 Kans. Additional synthesis and sorting steps can be added until large compound libraries are synthesized. To support the library production, the sorting of the individual compounds can be obtained following a final array of the reactors by sorting them into a spatially addressable MiniBlock for processing the cleavage [30]. A separate Cleavage Station permits the simultaneous cleavage of up to 96 compounds. Eventually, the compound can be collected by vacuum filtration in multiwell plates and subsequent centrifugation. It was previously shown that the IRORI technology can even be implemented in other data management and lab automation systems such as downstream liquid handling automation and drying systems as well as LC/MS, and MALDI-TOF-MS [30].

Here, we established the submonomer-based solid phase synthesis of a “proof of principle” CPPo library in IRORI MiniKans [31,32,33,34,35,36]. Eventually, the CPPo library was applied to HeLa cells for the screening of organelle targeting CPPos.

## 2. Results and Discussion

**Scheme 1:**
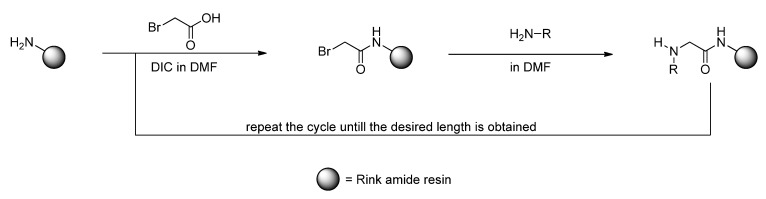
Sub-monomer approach to build up peptoids on solid supports.

For the synthesis of the polymer-supported CPPo library, the submonomer approach offers a preferable simple access (Scheme 1) [37,38]. As the name indicates, the *N-*substituted glycine building blocks (peptoid monomer) will be generated from two parts: Bromoacetic acid and a primary amine (submonomer). This method yields peptoids by consecutive coupling of bromoacetic acid (activated by DIC) and subsequent nucleophilic displacement of the bromine atom by a primary amine. This approach has the great advantage, that a variety of commercially available primary amines can be used directly to build up diverse peptoids. A protecting group strategy is only necessary if further reactive groups are present in the submonomer.

**Figure 2:**
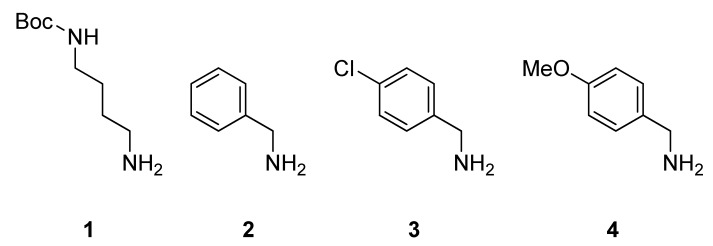
Featured submonomers **1**–**4**, which were used for the peptoid library.

The peptoid library was designed to consist of different tetramers. The synthesis was performed in a small scale split and mix approach. Two classes of submonomers (hydrophobic or hydrophilic, respectively) were used for the synthesis and each side chain could be either hydrophilic or hydrophobic. To make sure, that all peptoids bear at least one positive charge and thus can act as CPPos, the third position was chosen to be nonvariable and should always display a cationic and thus hydrophilic side chain. Having three variable positions for the permutation of hydrophobic and hydrophilic side chains available, the total library should therefore consists of 2^3^ = 8 peptoids. The variable three residues were even further diversified by different hydrophobic submonomers (Figure 2) to support the passage through the cell membrane. It has been shown for the corresponding CPPs that the polycationic and amphiphilic nature of the peptides supported either the membrane penetration or the cellular uptake in general [39,40]. Likewise, it has been shown that peptoids featuring multiple positively charged side chains were able to overcome the cell membrane [16,17,18,41]. All peptoids were conjugated with rhodamine B to be applicable to fluorescence microscopy, which allowed for monitoring the cellular uptake and intracellular distribution.

**Figure 3:**
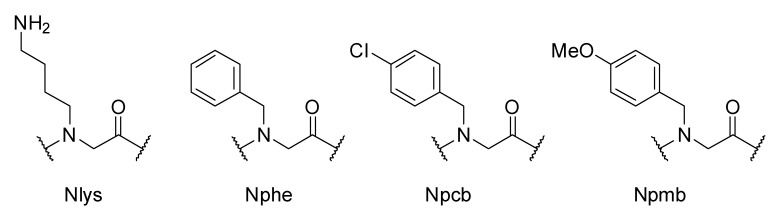
Annotation of the peptoid monomers. Nlys = *N*-4-aminobutylglycine, Nphe = *N*-benzylglycine, Npcb = *N*-(*p*-chlorobenzyl)glycine, Npmb = *N*-(*p*-methoxybenzyl)glycine.

The peptoid syntheses were performed with the mono-Boc-protected diamine **1** and three different benzylic amines **2**–**4**, which are depicted in Figure 2. After the acidic cleavage from the solid support, submonomer **1** was deprotected and provided a positive charge due to its protonation in aqueous media. Hydrophobicity was introduced by benzylic amines **2**–**4**. To distinguish between different isomeric peptoid sequences, a slightly different hydrophobic submonomer was used for each coupling step. We chose benzylamine (**2**), *p*-chlorobenzylamine (**3**) [42] and *p*-methoxybenzylamine (**4**) for this purpose to reveal Nphe, Npcb, and Npmb residues (Figure 3). Since the molecular masses of these hydrophobic submonomers were different, the actual sequence could be assigned by mass spectrometry. 

Bearing this in mind, we synthesized an eight-membered library of tetramers containing a three variable residues backbone and a nonvariable cationic side chain-containing residue at position three, coupled to a rhodamine B-fluorophore at the *N*-terminus. The resulting peptoids were annotated using a simple nomenclature, which is comparable to the common amino acid three letter code. To denote the peptoidic origin, the first letter of a peptoid monomer is always a capitalized N followed by an individual sequence of three small letters. The four codes, which were used in this article, are depicted in Figure 2. The abbreviations of the monomers Nlys and Nphe result from their analogy to the proteogenic amino acids lysine (Lys) and phenylalanine (Phe). In addition we used the abbreviation “RhoB” for the conjugated rhodamine B dye at the *N*-terminus. Following the peptide nomenclature, the peptoid sequences were also written from *N*- to *C*-terminus.

The tetrameric peptoids were assembled on polystyrene resin functionalized with the rink amide linker. The existing protocols of the well-known submonomer method were adjusted to an IRORI MiniKan-supported split and mix synthesis. To achieve this, the conditions were optimized for the synthesis of the homotetramer RhoB-Nlys_4_-NH_2_ (**5**). The final peptoid was verified by MALDI-TOF MS [43,44]. In general, the existing protocols of the submonomer method could be transferred to the reaction in the MiniKans by extending the reaction times. At first, the Fmoc-protected Rink amide resin was deprotected by incubating 3 × 10 min with 20% piperidine in DMF. We found complete reactions by using 1 M coupling solutions with incubation times of 90 min for the acylation and 2 h for the nucleophilic substitution with *N*-Boc-diaminobutane (**1**). The fluorophore rhodamine B was successfully coupled by overnight incubation with a 1 M solution of the dye, diisopropylcarbodiimide (DIC) and 1-hydroxybenzotriazol (HOBt). The final cleavage was achieved by incubating the resin with 95% trifluoroacetic acid (TFA) in dichloromethane. Due to the intensive color of the rhodamine B-labeled peptoids, the cleavage process could be optically monitored by repeated addition of fresh cleavage solution. The cleavage was completed after an incubation time of 2 h. The peptoid RhoB-Nlys_4_-NH_2_ (**5**) was simultaneously synthesized in three MiniKans to check the reliability of the conditions and to monitor statistical variations. Comparison of the HPLC chromatogram and the respective mass spectra of the triplicates revealed the recovery of the same product from all MiniKans. The crude product could be easily purified by HPLC with a purity of >99% with an average yield of 49%.

It should be mentioned that the shape and the size of the reaction vessel is very important with respect to the necessary volume of solvent. All the MiniKans should be entirely covered by the coupling/washing solutions to grant a preferably homogenous reaction. The vessel should allow for a prone orientation of the MiniKans. In order to save chemicals, the volume of the solvent should be just enough to submerge the MiniKans completely. If the volume has to be raised, the concentration of the coupling reagents should be kept constant to keep the reaction time short.

**Figure 4:**
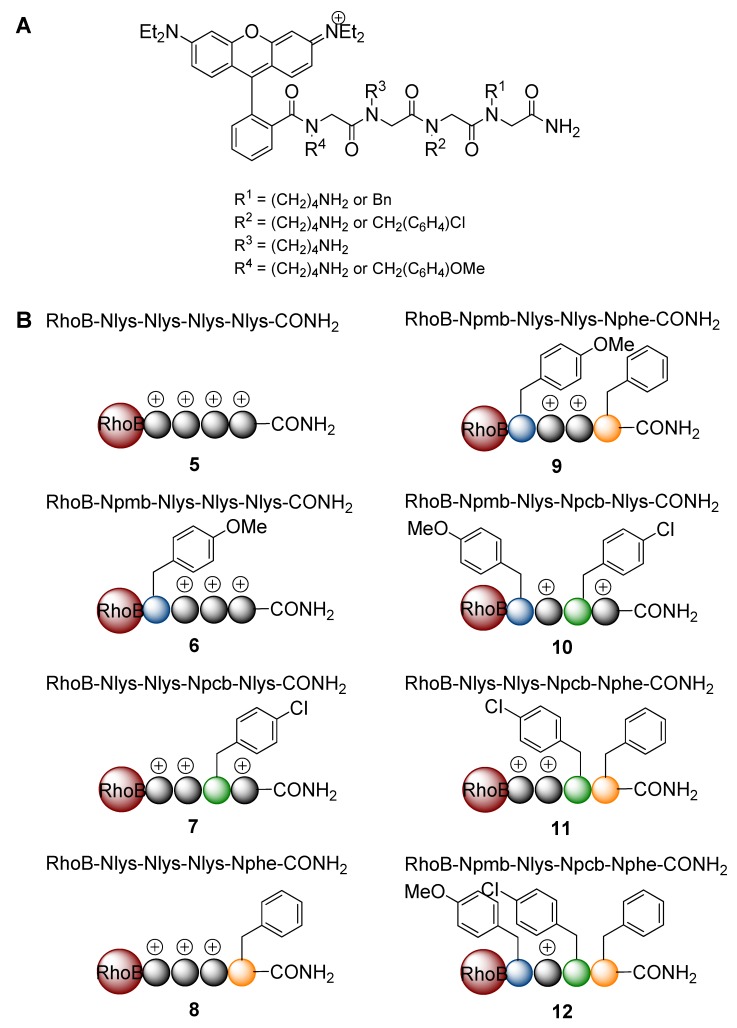
(**A**) General structure of the rhodamine B-labeled tetrameric peptoid library. (**B**) Schematic view of the library members. +, cationic Nlys residues; spheres are representing the peptoid backbone.

The combinatorial synthesis was conducted with 32 MiniKans to ensure the coverage of all possible permutations. Finally the library contained all eight peptoids between two and six times (Figure 4). The peptoids could be easily purified by HPLC to 82–99% purity. The *N*-Boc-diaminobutane (**1**) sub-monomer performed very well so that the Nlys homotetramer could be isolated in good yields. However, the *p*-methoxybenzylamine containing peptoids were synthesized with rather low yields (2.1–2.7%). This side chain is quite acid sensitive and leads therefore to degradation [45,46,47]. In general, the concept of MiniKan-based peptoid libraries proved to be compatible with the widely used submomomer approach and should thus be applicable to the synthesis of large and diverse libraries.

Eventually, all the purified peptoids were subjected to live cell imaging to screen for CPPos with organelle specificity. Subcellular or organelle-specific targeting is one of the major challenges in the delivery of bioactive molecules [48,49,50]. In particular, the targeting to mitochondria has lately emerged into focus of current research as mitochondria are involved in many metabolic and pathological processes [51,52,53,54]. Recently, synthetic mitochondria-penetrating peptides as special analogues of the CPPs have been developed [55,56,57,58,59,60]. The so-called mitochondria-penetrating peptides (MPPs) are identified as cationic but also lipophilic and their synthesis was rationally controlled and finally tuned for their application *in vivo* [55,56,57,58,59,60]. The herein reported library is also based on the rational to provide lipophilicity to the CPPos for mitochondrial targeting at least for some of the CPPos. 

**Figure 5:**
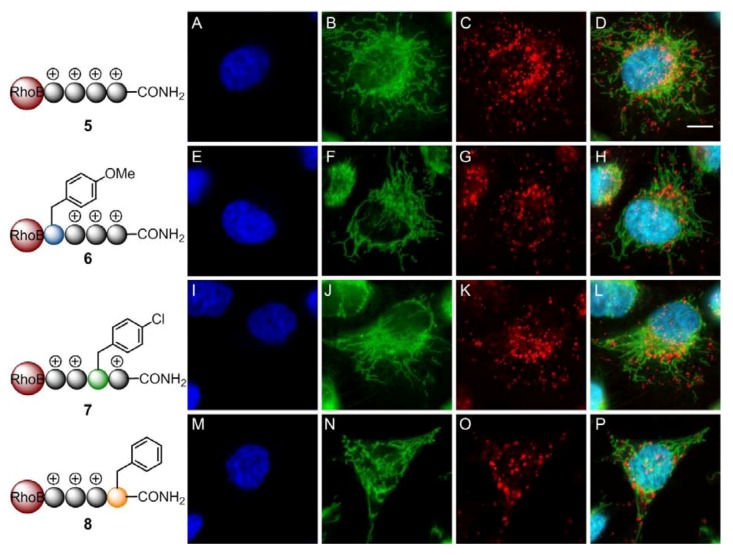
**CPPos with endosomal localization**. Cellular uptake of endosomal CPPos **5**–**8** in HeLa cells. 1 × 10^4^ HeLa wt cells were treated with 1 µM of **5** (**A**–**D**), **6** (**E**–**H**), **7** (**I**–**L**), and **8** (**M**–**P**) for 24 h at 37 °C. For costaining of the nuclei and the mitochondria the cells were treated with Hoechst 33342 (blue: **A**, **E**, **I**, **M**) and 100 nM Mitochondria Green^TM^ (green: **B**, **F**, **J**, **N**). Eventually the cells were analyzed by fluorescence confocal imaging. The images **D**, **H**, **L**, **P** show the merges of the respective emission channels of each line by using the following PMTs for the emission: 417–468 nm for the detection of the nuclei (blue: **A**, **E**, **I**, **M**), 499–552 nm for the detection of the mitochondria (green: **B**, **F**, **J**, **N**), and 593–696 nm for the detection of the rhodamine B labeled peptoids (red: **C**, **F**, **K**, **O**). Scale bar = 10 µm.

**Figure 6:**
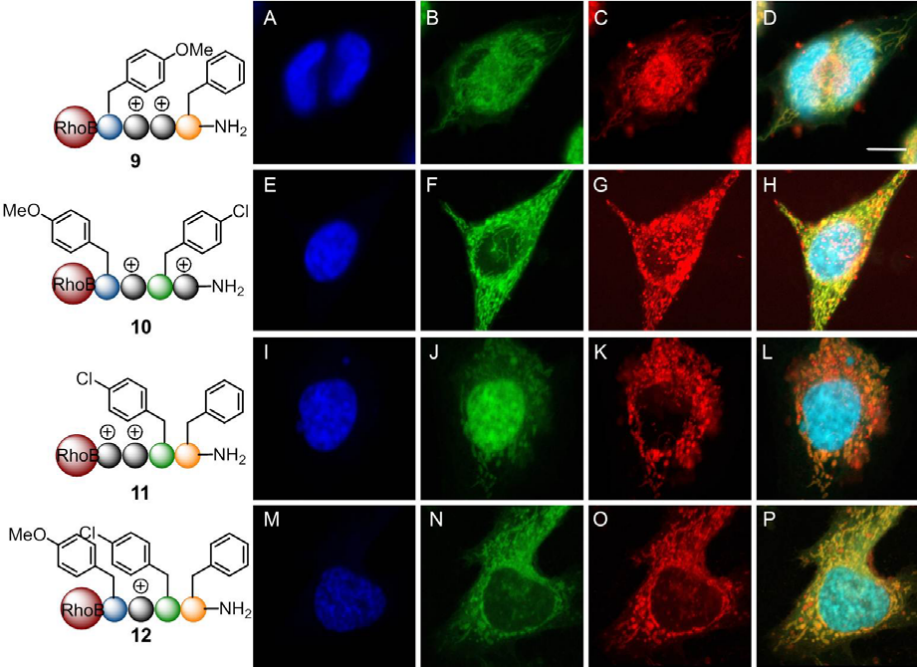
**CPPos with mitochondrial localization**. Cellular uptake of mitochondrial CPPos **9**–**12** in HeLa cells. 1 × 10^4^ HeLa wt cells were treated with 1 µM of **9** (**A**–**D**), **10 **(**E**–**H**), **11** (**I**–**L**), and **12** (**M**–**P**) for 24 h at 37 °C. For co-staining of the nuclei and the mitochondria the cells were treated with Hoechst 33342 (blue: **A**, **E**, **I**, **M**) and 100 nM Mitochondria Green^TM^ (green: **B**, **F**, **J**, **N**). Eventually, the cells were analyzed by fluorescence confocal imaging. The images **D**, **H**, **L**, **P** show the merges of the respective emission channels of each line by using the following PMTs for the emission: 417–468 nm for the detection of the nuclei (blue: **A**, **E**, **I**, **M**), 499–552 nm for the detection of the mitochondria (green: **B**, **F**, **J**, **N**), and 593–696 nm for the detection of the rhodamine B labeled peptoids (red: **C**, **F**, **K**, **O**). Scale bar = 10 µm.

HeLa cells were treated with 1 and 10 µM of the corresponding peptoids for 1 and 24 h. Uptake was already observed for both concentrations after 1 h. However, for the better visualization of the organellar accumulation the incubation period was prolonged to 24 h. Co-localization experiments were performed by co-staining of the mitochondria with the mitochondrial marker Mitotracker Green^TM^ (Figure 5 and Figure 6). All potential CPPos were efficiently taken up by the cells (Figure 5). As expected from our previous findings for all cationic CPPos [16,17], low concentration of the sequence RhoB-Nlys_4_-NH_2_ (**5**) revealed an endosomal/lysosomal staining (Figure 5A–D). At higher concentrations it was released into the cytosol and the nucleus. The uptake was comparable to that observed with the respective CPPs.

Further comparison of the uptake of the other members of the library showed an endosomal/lysosomal accumulation and subsequent release into the cytosol and nucleus for the CPPos **6**–**8** containing 3 Nlys residues (Figure 5**E**–**H**,**I**–**L**,**M**–**P**). By exchanging a further Nlys residue with a residue containing a lipophilic side chain as in CPPos **9**–**12**, the intracellular accumulation was profoundly different, while the uptake was still strong (Figure 6).

By colocalizing the peptides with the mitochondrial marker Mitotracker Green^TM^ Pearson’s correlation coefficients (Rr) of the corresponding fluorescence profiles could be calculated reflecting mitochondrial specificity. While the latter CPPo sequences **5-8** with 3-4 Nlys residues were excluded from the mitochondria (Pearson correlation coefficients: Rr(**5**) = −0.22; Rr(**6**) = −0.16; Rr(**7**) = −0.10; Rr(**8**) = −0.11), sequences with only 2 Nlys side chains **9**–**11** exhibit at least a partial localization with the mitochondria (Rr(**9**) = +0.16; Rr(**10**) = +0.47; Rr(**11**) = +0.68; Rr(**12**) = +0.86). However, they also exhibit an additional accumulation within the endosomal compartment with increasing concentrations. CPPo **12** containing only one Nlys residue displayed a very high level of mitochondrial colocalization with a Pearson coefficient of Rr = +0.86, while the CPPos with two remaining Nlys residues were a little less abundant in the mitochondria (Rr = +0.16 – +0.68). Notably, there were even subtle differences in the mitochondrial colocalization of the CPPos containing only 2 Nlys residues. By alternating the Nlys residues with lipophilic residues as well as by enhancing the lipophilicity at the *C-*terminus one can trigger mitochondrial colocalization of the CPPos **10** and **11** (Figure 6**E**–**F**,**I**–**L**). 

This indicated that minor changes to the chemical structure of the CPPos could have a significant impact on the organellar targeting of the transport moieties. Cationic tetrameric CPPos with a net charge of +3 and higher will be localized to the endosomal compartment with a final release into the cytosol. Electrostatic interactions play a significant role in the membrane affinity of those cationic moieties. With a pK_a_ of 10.4, the side chains of the peptoids display a similar pK_a_ as lysine. In aqueous solution the CPPo will be highly charged as the amino groups are protonated and transformed into NH^3+^-groups giving a total charge of the molecule of +3 up to +4. By interacting with the negatively charged proteoglycans at the plasma membrane these CPPos can be attracted close to the lipid bilayer to be subsequently engulfed with the plasma membrane and endocytosed. If a cationic tetrametric CPPo with +2 charge should be routed to the mitochondria it must possess sufficient lipophilicity such as displayed in CPPos **9–11**. The CPPo **12 **containing charge of +1 and lipophilic side chains will always be localized in the mitochondria. As it was already described for the mitochondria penetrating peptides (MPPs) [56,57], there seems to be a well-defined lipophilicity threshold that is required for mitochondrial routing. So far, there were no obvious localization effects of the *p*-methoxy and *p-*chloro substituents on the lipophilic residues, although CPPo **9**, which did not contain any Npcb residues was the weakest mitochondrial transporter. However, the IRORI system has to be exploited for more diverse libraries in the future to allow for a more detailed study of the determinants of mitochondrial and other organelle specific transport. 

## 3. Experimental

### 3.1. General Remarks

All reactions were performed at room temperature in IRORI MiniKans on Rink amide resin (0.64 mmol/g, 50 mg per MiniKan). The volume of the different washing or coupling solutions was constant during the entire synthesis (3 mL per MiniKan). The coupling reagents had a concentration of 1 M each. After each step described below, the MiniKans were filtered from the solution. After each transformation the resin was thoroughly washed with 2 × DMF and 1 × DMF biograde. The washing steps were performed by stirring with the washing solution for 10 min. MALDI-TOF mass spectra of the peptoids were obtained by using a Bruker Biflex IV spectrometer with a pulsed ultraviolet nitrogen laser, 200 µJ at 337 nm and a time-of-flight mass analyzer with 125 cm linear flight path. Reversed phase analytical HPLC was performed (Agilent Series 1200, using a C18 PerfectSil Target column MZ Analytik, 3–5 µm, 4.0 × 250 mm; Flow rate: 1 mL/min; solvent A: 0.1% TFA in water; solvent B: 0.1% TFA in acetonitrile). Reversed phase preparative HPLC was performed using a JASCO HPLC system, using a C18 Vydac 218TP Series column (Grace Davison Discovery Sciences, 10 µm, 22 × 250 mm). Flow rate: 15 mL/min; solvent A: 0.1% TFA in water; solvent B: 0.1% TFA in acetonitrile. 

### 3.2. Sub-Monomer Synthesis

The synthesis of *tert*-butyl (4-aminobutyl)carbamate (**1**) was carried out as reported by Schröder *et al.* [16].

### 3.3. Synthesis of the Peptoid Library by using IRORI MiniKans (Data are given for 32 MiniKans in a 300 mL Erlenmeyer Flask)

Fmoc-protected Rink amide resin was loaded into IRORI MiniKans. At first, the resin was swollen in DMF for 1 h. Afterwards the resin was washed with 3 × DMF. The Fmoc deprotection was carried out by stirring with 3 × piperidine (20% in DMF) for 10 min. Subsequent acylation was performed by reaction with bromoacetic acid and diisopropyl-carbodiimide (DIC) in DMF biograde for 90 min. For the conjugation with either a hydrophilic or a hydrophobic submonomer, the MiniKans were randomly split in two portions. One portion of the MiniKans was stirred in a solution of the hydrophilic submonomer, whereas the other portion was stirred in a solution of the hydrophobic submonomer (except for the third coupling where just the hydrophilic submonomer was used). Afterwards, all MiniKans were combined (mixed) again (split and mix method) [61]. This sequence of acylation and nucleophilic substitution by a sub-monomer was repeated four times in total. The hydrophilic submonomer **1** was used for every nucleophilic substitution, whereas the hydrophobic submonomers **2**, **3** and **4** were used for the first, second and fourth nucleophilic substitution, respectively. The submonomers were dissolved in DMF biograde and the resin was incubated for 2 h. After the synthesis of the tetramer, rhodamine B was coupled by activating with DIC and 1-hydroxybenzotriazole hydrate (HOBt) in DMF biograde through overnight incubation. Finally, the peptoid of each MiniKan was cleaved separately by adding trifluoroacetic acid (95% in CH_2_Cl_2_, 5 mL) and incubating for 2 h. The cleavage solution was filtered and the resin was thoroughly washed with MeOH until the washing solution remained colorless. The solvent was removed under reduced pressure and the crude product was dissolved in H_2_O/acetonitrile (2:1) and subsequently lyophilized. The library contained every possible permutation of the tetrameric peptoid (2–6 times). The different peptoids were purified by RP-HPLC. All resulted peptoids were analyzed by MALDI-TOF MS [43,44]. The data below are related to one MiniKan approach for each peptoid.

*RhoB-Nlys_4_-NH_2_* (**5**): 23.8 mg (49%, HPLC purity: >99%). MALDI-TOF MS: *m/z*: 954 [M]^+^.

*RhoB-Npmb-Nlys_3_-NH_2_* (**6**): 1.18 mg (2.5%, HPLC purity: 83%). MALDI-TOF MS: *m/z*: 1003 [M]^+^.

*RhoB-Nlys_3_-Nphe-NH_2_* (**7**): 10.3 mg (23%, HPLC purity: 90%). MALDI-TOF MS: *m/z*: 973 [M]^+^.

*RhoB-Nlys_2_-Npcb-Nlys-NH_2_* (**8**): 2.67 mg (5.7%, HPLC purity: 88%). MALDI-TOF MS: *m/z*: 1007 [M]^+^.

*RhoB-Npmb-Nlys_2_-Nphe-NH_2_* (**9**): 1.16 mg (2.7%, HPLC purity: 85%). MALDI-TOF MS: *m/z*: 1022 [M]^+^.

*RhoB-Npmb-Nlys-Npcb-Nlys-NH_2_* (**10**): 1.01 mg (2.3%, HPLC purity: 83%). MALDI-TOF MS: *m/z*: 1057 [M]^+^.

*RhoB-Npmb-Nlys_3_-NH_2_* (**11**): 1.18 mg (2.5%, HPLC purity: 83%). MALDI-TOF MS: *m*/*z*: 1003 [M]^+^.

*RhoB-Npmb-Nlys-Npcb-Nphe-NH_2_* (**12**): 0.89 mg (2.1%, HPLC purity: 82%). MALDI-TOF MS: *m/z*: 1076 [M]^+^.

### 3.4. Cell Culture Techniques for Mammalian Cells

All procedures with mammalian cells were carried out under sterile conditions. 1 × 10^4^ HeLa (human cervix carcinoma) cells were plated into each well of a 8-well µslide from IBIDI (Ibitreat, Martinsried, Germany) and cultured in 200 µL Dulbecco’s modified Eagle’s medium, high glucose, (DMEM, Invitrogen, Karlsruhe, Germany) supplemented with 10% fetal calf serum (FCS, PAA) and1 U/mL penicillin/streptomycin at 37 °C, 5% CO_2_.

### 3.5. Treatment of HeLa Cells with Rhodamine B Coupled Peptoids

The purified peptoids were dissolved in bidistilled water to yield a 2 mM stock solution and were further diluted with 10% DMEM to yield the respective incubation media. The cells were cultured as described above were incubated with the different peptoids at final concentrations of 1–10 µM. Cellular uptake of the peptoids was measured by live-cell imaging after 1 and 24 h as fixation would alter the intracellular distribution as described for other polycationic species.

### 3.6. Live Imaging by Confocal Microscopy

Visualization of the peptoids was achieved by confocal microscopy using a Leica TCS-SP5 II, equipped with a DMI6000 microscope. The peptoids were excited at 561 nm using a DPSS laser. The Mitotracker Green (Invitrogen, Karlsruhe, Germany) for the detection of the mitochondria was excited at 488 nm using an argon laser and the Hoechst 33342 for the detection of the nuclei was excited at 364 nm using a UV laser. The objective was a HCX PL APO CS 63.0 × 1.2 Water UV. The exposure was set to minimize oversaturated pixels in the final images. Fluorescence emission was measured at 417–468 nm for the detection of the nuclei, at 499–552 nm for the detection of the mitochondria, and 593–696 nm for the detection of the rhodamine B labeled peptoids. Image acquisition was conducted at a lateral resolution of 1,024 × 1,024 pixels and 8 bit depth using LAS-AF 2.0.2.4647 acquisition software.

## 4. Conclusions

In this study we established the submonomer-based solid phase synthesis of a “proof of principle” CPPo library in IRORI MiniKans to expand the amount of peptoids for future phenotypic high throughput screens. Although the split and mix process of the MiniKans was performed manually due to the size of the “proof of principle” CPPo library, the IRORI system has many advantages. This technology allows for the straightforward synthesis, facile derivatization, barcoding and upscaling of large and diverse libraries. The synthesis of diverse oligomeric libraries that display a variety of backbone scaffolds and side-chain appendages are a very promising source of novel CPPos, which can be used to either target different cellular organelles or even different tissues and organs. By the introduction of different lipophilic residues mixed with cationic moieties we have engineered a class of cell-penetrating peptoids that efficiently enter human cells but differed in cellular uptake and organelle localization. While highly charged CPPos enter the cells by endocytosis with a subsequent endosomal release, critical levels of lipophilicity allow other CPPos to specifically localize to mitochondria once a certain lipophilicity threshold is reached. In future experiments the IRORI approach will be expanded to generate larger libraries for the determination of the exact requirements that impart mitochondrial or other organelle localization to lipophilic cations, and may provide a means to engineer the cellular trafficking of bioactive compounds as it was shown for CPPs.

## References

[1] Wadia J.S., Dowdy S.F. (2002). Protein transduction technology. Curr. Opin. Biotechnol..

[2] Vives E., Richard J.P., Rispal C., Lebleu B. (2003). TAT peptide internalization: Seeking the mechanism of entry. Curr. Protein. Pept. Sci..

[3] Simon R.J., Kania R.S., Zuckermann R.N., Huebner V.D., Jewell D.A., Banville S., Ng S., Wang L., Rosenberg S., Marlowe C.K. (1992). Peptoids: A modular approach to drug discovery. Proc. Natl. Acad. Sci. USA.

[4] Peretto I., Sanchez-Martin R.M., Wang X.H., Ellard J., Mittoo S., Bradley M. (2003). Cell penetrable peptoid carrier vehicles: Synthesis and evaluation. Chem. Commun..

[5] Sawant R., Torchilin V. (2010). Intracellular transduction using cell-penetrating peptides. Mol. Biosyst..

[6] Ezzat K., Zaghloul E.M., El Andaloussi S., Lehto T., El-Sayed R., Magdy T., Smith C.I., Langel U. (2012). Solid formulation of cell-penetrating peptide nanocomplexes with siRNA and their stability in simulated gastric conditions. J. Control. Release.

[7] Holm T., Raagel H., Andaloussi S.E., Hein M., Mae M., Pooga M., Langel U. (2011). Retro-inversion of certain cell-penetrating peptides causes severe cellular toxicity. Biochim. Biophys. Acta.

[8] Yin H., Moulton H.M., Betts C., Seow Y., Boutilier J., Iverson P.L., Wood M.J. (2009). A fusion peptide directs enhanced systemic dystrophin exon skipping and functional restoration in dystrophin-deficient mdx mice. Hum. Mol. Genet..

[9] Umezawa N., Gelman M.A., Haigis M.C., Raines R.T., Gellman S.H. (2002). Translocation of a beta-peptide across cell membranes. J. Am. Chem. Soc..

[10] Nakase I., Takeuchi T., Tanaka G., Futaki S. (2008). Methodological and cellular aspects that govern the internalization mechanisms of arginine-rich cell-penetrating peptides. Adv. Drug Deliv. Rev..

[11] Gademann K., Hintermann T., Schreiber J.V. (1999). Beta-peptides: Twisting and turning. Curr. Med. Chem..

[12] Murphy J.E., Uno T., Hamer J.D., Cohen F.E., Dwarki V., Zuckermann R.N. (1998). A combinatorial approach to the discovery of efficient cationic peptoid reagents for gene delivery. Proc. Natl. Acad. Sci. USA.

[13] Uno T., Beausoleil E., Goldsmith R.A., Levine B.H., Zuckermann R.N. (1999). New submonomers for poly N-substituted glycines (peptoids). Tetrahedron Lett..

[14] Wender P.A., Mitchell D.J., Pattabiraman K., Pelkey E.T., Steinman L., Rothbard J.B. (2000). The design, synthesis, and evaluation of molecules that enable or enhance cellular uptake: Peptoid molecular transporters. Proc. Natl. Acad. Sci. USA.

[15] Wright L.R., Rothbard J.B., Wender P.A. (2003). Guanidinium rich peptide transporters and drug delivery. Curr. Prot. Pept. Sci..

[16] Schröder T., Schmitz K., Niemeier N., Balaban T.S., Krug H.F., Schepers U., Bräse S. (2007). Solid-phase synthesis, bioconjugation, and toxicology of novel cationic oligopeptoids for cellular drug delivery. Bioconjug. Chem..

[17] Schröder T., Niemeier N., Afonin S., Ulrich A.S., Krug H.F., Bräse S. (2008). Peptoidic amino- and guanidinium-carrier systems: targeted drug delivery into the cell cytosol or the nucleus. J. Med. Chem..

[18] Eggenberger K., Birtalan E., Schröder T., Bräse S., Nick P. (2009). Passage of Trojan peptoids into plant cells. ChemBioChem.

[19] Rudat B., Birtalan E., Thomé I., Kölmel D.K., Horhoiu V.L., Wissert M.D., Lemmer U., Eisler H.J., Balaban T.S., Bräse S. (2010). Novel pyridinium dyes that enable investigations of peptoids at the single-molecule level. J. Phys. Chem. B.

[20] Birtalan E., Rudat B., Kölmel D.K., Fritz D., Vollrath S.B.L., Schepers U., Bräse S. (2011). Investigating rhodamine B-Labeled peptoids: Scopes and limitations of its applications. Biopolymers.

[21] Lee M.M., French J.M., Disney M.D. (2011). Influencing uptake and localization of aminoglycoside-functionalized peptoids. Mol. Biosyst..

[22] Seo J., Ren G., Liu H., Miao Z., Park M., Wang Y., Miller T.M., Barron A.E., Cheng Z. (2012). *In Vivo* biodistribution and small animal PET of (64)Cu-Labeled antimicrobial peptoids. Bioconjug. Chem..

[23] Huang W., Seo J., Lin J.S., Barron A.E. (2012). Peptoid transporters: Effects of cationic, amphipathic structure on their cellular uptake. Mol. Biosyst..

[24] Tan N.C., Yu P., Kwon Y.U., Kodadek T. (2008). High-throughput evaluation of relative cell permeability between peptoids and peptides. Bioorg. Med. Chem..

[25] Aditya A., Kodadek T. (2012). Incorporation of heterocycles into the backbone of peptoids to generate diverse peptoid-inspired one bead one compound libraries. ACS Comb. Sci..

[26] Diaz-Mochon J.J., Fara M.A., Sanchez-Martin R.M., Bradley M. (2008). Peptoid dendrimers-microwave-assisted solid-phase synthesis and transfection agent evaluation. Tetrahedron Lett..

[27] Fara M.A., Diaz-Mochon J.J., Bradley M. (2006). Microwave-assisted coupling with DIC/HOBt for the synthesis of difficult peptoids and fluorescently labelled peptides—a gentle heat goes a long way. Tetrahedron Lett..

[28] Li S., Bowerman D., Marthandan N., Klyza S., Luebke K.J., Garner H.R., Kodadek T. (2004). Photolithographic synthesis of peptoids. J. Am. Chem. Soc..

[29] Olivos H.J., Alluri P.G., Reddy M.M., Salony D., Kodadek T. (2002). Microwave-assisted solid-phase synthesis of peptoids. Org. Lett..

[30] Goodman B. (1999). Managing the workflow of a high-throughput organic synthesis laboratory: A marriage of automation and information management technologies. J. Lab. Automation.

[31] Cheung A.W., Qi L., Gore V., Chu X.J., Bartkovitz D., Kurylko G., Swistok J., Danho W., Chen L., Yagaloff K. (2005). Preparation of human Melanocortin-4 receptor agonist libraries: Linear peptides X-Y-DPhe7-Arg8-Trp(or 2-Nal)9-Z-NH2. Bioorg. Med. Chem. Lett..

[32] Xiao X.Y., Li R.S., Zhuang H., Ewing B., Karunaratne K., Lillig J., Brown R., Nicolaou K.C. (2000). Solid-phase combinatorial synthesis using MicroKan reactors, Rf tagging, and directed sorting. Biotechnol. Bioeng..

[33] Nicolaou K.C., Xiao X.Y., Parandoosh Z., Senyei A., Nova M.P. (1995). Radiofrequency encoded combinatorial chemistry. Angew. Chem. Int. Ed..

[34] Nagai K., Doi T., Sekiguchi T., Namatame I., Sunazuka T., Tomoda H., Omura S., Takahashi T. (2006). Synthesis and biological evaluation of a beauveriolide analogue library. J. Comb. Chem..

[35] Nicolaou K.C., Vourloumis D., Li T.H., Pastor J., Winssinger N., He Y., Ninkovic S., Sarabia F., Vallberg H., Roschangar F. (1997). Designed epothilones: Combinatorial synthesis, tubulin assembly properties, and cytotoxic action against taxol-resistant tumor cells. Angew. Chem. Int. Ed..

[36] Marsault E., Hoveyda H.R., Peterson M.L., Gagnon R., Vezina M., Pinault J.F., Landry A., Saint-Louis C., Ouellet L.G., Beauchemin S. (2009). High throughput solid phase parallel synthesis of macrocyclic peptidomimetics. Adv. Exp. Med. Biol..

[37] Zuckermann R.N., Kerr J.M., Kent S.B.H., Moos W.H. (1992). Efficient method for the preparation of peptoids [Oligo(N-Substituted Glycines)] by submonomer solid-phase synthesis. J. Am. Chem. Soc..

[38] Culf A.S., Ouellette R.J. (2010). Solid-phase synthesis of N-substituted glycine oligomers (alpha-peptoids) and derivatives. Molecules.

[39] Goun E.A., Shinde R., Dehnert K.W., Adams-Bond A., Wender P.A., Contag C.H., Franc B.L. (2006). Intracellular cargo delivery by an octaarginine transporter adapted to target prostate cancer cells through cell surface protease activation. Bioconjug. Chem..

[40] Wender P.A., Jessop T.C., Pattabiraman K., Pelkey E.T., VanDeusen C.L. (2001). An efficient, scalable synthesis of the molecular transporter octaarginine via a segment doubling strategy. Org. Lett..

[41] Schröder T., Quintilla A., Setzler J., Birtalan E., Wenzel W., Bräse S. (2007). Joint experimental and theoretical investigation of the propensity of peptoids as drug carriers. WSEAS Trans. Biol. Biomed..

[42] Chen C.L., Qi J., Zuckermann R.N., DeYoreo J.J. (2011). Engineered biomimetic polymers as tunable agents for controlling CaCO3 mineralization. J. Am. Chem. Soc..

[43] Thakkar A., Cohen A.S., Connolly M.D., Zuckermann R.N., Pei D. (2009). High-throughput sequencing of peptoids and peptide-peptoid hybrids by partial edman degradation and mass spectrometry. J. Comb. Chem..

[44] Simpson L. S., Kodadek T. (2012). A cleavable scaffold strategy for the synthesis of one-bead one-compound cyclic peptoid libraries that can be sequenced by tandem mass spectrometry. Tetrahedron Lett..

[45] Aquino C., Sarkar M., Chalmers M.J., Mendes K., Kodadek T., Micalizio G.C. (2012). A biomimetic polyketide-inspired approach to small-molecule ligand discovery. Nat. Chem..

[46] Hooks J.C., Matharage J.P., Udugamasooriya D.G. (2011). Development of homomultimers and heteromultimers of lung cancer-specific peptoids. Biopolymers.

[47] Klimkait T., Felder E.R., Albrecht G., Hamy F. (1998). Rational optimization of a HIV-1 Tat inhibitor: rapid progress on combinatorial lead structures. Biotechnol. Bioeng..

[48] D'Souza G.G., Cheng S.M., Boddapati S.V., Horobin R.W., Weissig V. (2008). Nanocarrier-assisted sub-cellular targeting to the site of mitochondria improves the pro-apoptotic activity of paclitaxel. J. Drug Target..

[49] Boddapati S.V., D'Souza G.G., Erdogan S., Torchilin V.P., Weissig V. (2008). Organelle-targeted nanocarriers: Specific delivery of liposomal ceramide to mitochondria enhances its cytotoxicity *in vitro* and *in vivo*. Nano Lett..

[50] Weissig V. (2005). Targeted drug delivery to mammalian mitochondria in living cells. Expert Opin. Drug. Deliv..

[51] Wallace D.C. (1999). Mitochondrial diseases in man and mouse. Science.

[52] Chinnery P.F., Turnbull D.M. (2000). Mitochondrial DNA mutations in the pathogenesis of human disease. Mol. Med. Today.

[53] Wallace D.C. (2005). Mitochondria and cancer: Warburg addressed. Cold Spring Harb. Symp. Quant. Biol..

[54] Wallace D.C. (2005). A mitochondrial paradigm of metabolic and degenerative diseases, aging, and cancer: A dawn for evolutionary medicine. Annu. Rev. Genet..

[55] Horton K.L., Kelley S.O. (2009). Engineered apoptosis-inducing peptides with enhanced mitochondrial localization and potency. J. Med. Chem..

[56] Horton K.L., Stewart K.M., Fonseca S.B., Guo Q., Kelley S.O. (2008). Mitochondria-penetrating peptides. Chem. Biol..

[57] Horton K.L., Pereira M.P., Stewart K.M., Fonseca S.B., Kelley S.O. (2012). Tuning the activity of mitochondria-penetrating peptides for delivery or disruption. ChemBioChem.

[58] Kelley S.O., Stewart K.M., Mourtada R. (2011). Development of novel peptides for mitochondrial drug delivery: Amino acids featuring delocalized lipophilic cations. Pharm. Res..

[59] Yousif L.F., Stewart K.M., Horton K.L., Kelley S.O. (2009). Mitochondria-penetrating peptides: sequence effects and model cargo transport. ChemBioChem.

[60] Yousif L.F., Stewart K.M., Kelley S.O. (2009). Targeting mitochondria with organelle-specific compounds: Strategies and applications. ChemBioChem.

[61] Furka A., Sebestyen F., Asgedom M., Dibo G. (1991). General method for rapid synthesis of multicomponent peptide mixtures. Int. J. Pept. Protein Res..

